# Reply to: Testing the adaptive hypothesis of lagging-strand encoding in bacterial genomes

**DOI:** 10.1038/s41467-022-30014-2

**Published:** 2022-05-12

**Authors:** Houra Merrikh, Christopher Merrikh

**Affiliations:** Department of Biochemistry, Vanderbilt University, Nashville, TN USA

**Keywords:** Molecular evolution, Genome evolution, Evolutionary genetics, Bacterial genomics

**replying to** Haoxuan Liu et al. *Nature Communications* 10.1038/s41467-022-30000-8 (2022)

Several previous studies by our group suggest that positive selection can drive certain (not all) genes to be retained in the lagging-strand orientation^1–3^. This is likely the result of multiple factors including accelerated evolution through replication-transcription conflicts^1–4^. Liu and Zhang challenge this view, and claim that the GC Skew-based method we used for detecting gene inversions is flawed^4^. Though the GC skew method does have a detection limit, we provide new evidence that the fundamental assumptions of our model, and our general conclusions, are accurate. We also introduce technical changes that improve the sensitivity of our method. The resulting high resolution data closely agree with Liu and Zhang’s phylogeny-based gene inversion data, and indicate that the trends we originally identified are stronger than they initially appeared: Across species, 89–96% of lagging-strand genes appear to be native leading strand genes that changed orientation. Our statistical analyses offer further support for the notion that for some genes, the lagging-strand orientation can be adaptive.

Inconsistent vocabulary is a source of confusion in this debate. Liu and Zhang’s phylogeny-based method identifies conserved orthologs that changed orientation during the divergence of an ancestral and descendant species. Liu and Zhang refer to all such events as “inversions” in accordance with common usage. However, this is distinct from the definition we used^1^. For clarity, here we refer to all change-of-orientation events as “flips”. There are two flip subtypes: The first results in a gene with a negative GC skew (Fig. 1, upper graphs). We originally called these “inversions”. However, for clarity we now call them “GC skew inversions”. We interpret the negative GC skew as an indication that the gene in question has physically flipped from its typical orientation, established over long-term evolution, to the opposing orientation. In the second sub-type, the flip results in a positive GC skew (Fig. 1, lower graphs). We describe these events as “GC skew reversions” as they reproduce the standard positive GC skew observed across the chromosome, and presumably restore a gene to its typical orientation. Our method cannot, and was never intended to identify GC skew reversions. Thus, our paper’s “inversions” (GC skew inversions) should represent only a subset of Liu and Zhang’s “inversions” (pooled GC skew reversions and inversions).

**Fig. 1:**
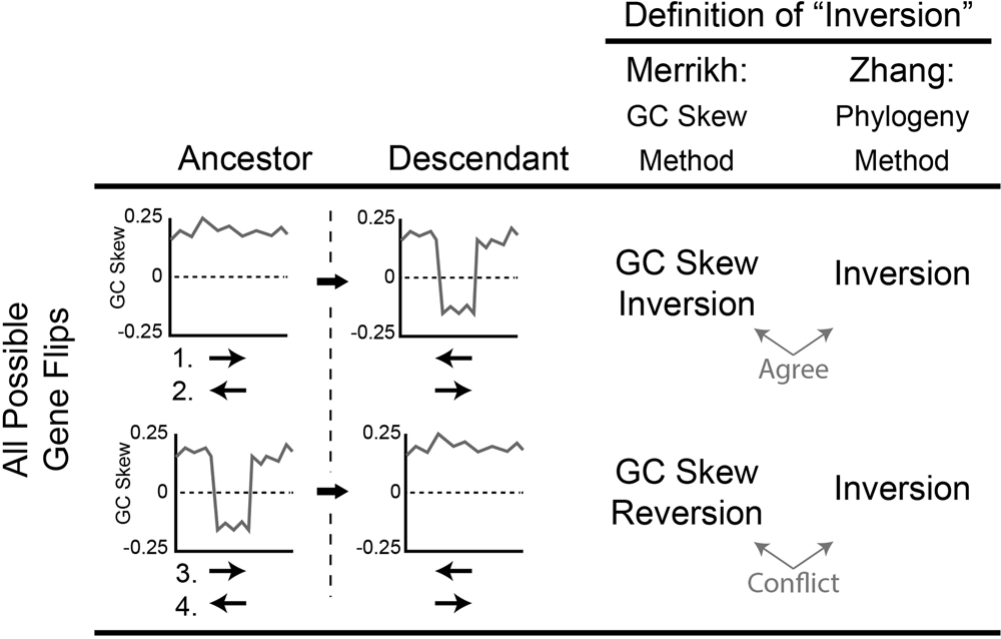
Comparison of gene “inversion” definitions and detection methods. The two studies use different definitions of “gene inversion”. For clarity, we use “Gene Flip” to mean any change-of-orientation event. Gene flips have two subtypes resulting in a gene having a negative GC Skew (upper graphs) or a positive GC skew (lower graphs). The Merrikh and Zhang definitions agree in the first scenario (upper graphs), but conflict in the second (lower graphs). As both gene orientation and GC skew sign (±) are important considerations, there are four possible circumstances (numbered 1–4) indicated under the GC skew plots. In case 1, a natively leading strand gene (indicated by the positive GC skew) undergoes a flip resulting in a GC skew inversion. In 2, a natively lagging-strand gene undergoes a flip, also resulting in a GC skew inversion. In 3, a leading strand gene with a negative GC skew flips, resulting in a GC skew reversion. In 4, a lagging-strand gene with a negative GC skew flips, resulting a GC skew reversion.

To identify inconsistent data points between the two studies, we calculated the GC skew for each of the ancestor/descendant orthologues Liu and Zhang identified (this manuscript’s Supplementary Data 1, 2, 3). These data show that, as expected, many flips are GC skew inversions, while others are GC skew reversions. This confirms that the differences between Liu and Zhang’s data and our own^4^ are entirely appropriate. These data also show that most “false negative errors” are simply GC skew reversion events. For example, in the *M. penetrans*/*M. gallisepticum* comparison, 27/52 flips are GC skew inversions and 22/52 are reversions, thus explaining 88% (22/25 total) of the perceived errors. (The remaining discrepancies are discussed below.) Notably, Liu and Zhang report a particularly low agreement between the two methods for lagging-to-leading strand flips. The data show that this is because most lagging-to-leading strand gene flips are GC skew reversions (Supplementary Data 1, 2, 3).

Liu and Zhang suggest improving our method’s accuracy by analyzing only 3rd codon position nucleotides (CP3), rather than whole-gene sequences when calculating the GC skew. We hypothesized that CP3 should be the least reliable source of information because these bases can mutate without significant consequence. Therefore, after a flip that results in a GC skew inversion, the CP3-based GC skew value should rapidly rise, preventing detection of the orientation change. We also note that the positive GC skew of whole chromosome arms is clearly detected using a sliding window which completely ignores codon position^5^. As such, it should be unnecessary or even counterproductive to examine only CP3 nucleotides. Nevertheless, we tested Liu and Zhang’s hypothesis by calculating the GC skew using either the whole-gene sequence, or only nucleotides in the 1st, 2nd, or 3rd codon positions (Supplementary Data 4). We observe good agreement between the whole-gene and the codon positions 1 and 2-based data. However, as predicted, there is lower agreement with the 3rd codon position-based data, especially for lagging-strand genes (Supplementary Data 4, Fig. 5 in ref. ^1^). As we previously showed, the lagging strand is enriched in GC skew inversions, explaining the latter observation^4^. Hence the 3rd codon position-based GC skew calculation is the lowest fidelity method for GC skew inversion detection.

Interestingly, both CP1 and CP3-based GC skew values are generally positive, matching the trend across the chromosome (Fig. 2, Supplementary Data 4). In fact, the magnitude of the GC skew is greatest for CP1 nucleotides (Fig. 2, top graphs). As CP1 nucleotides are under far higher selection pressure than CP3 nucleotides, these data suggest that GC skew values are more strongly influenced by replication-related mutation bias than selection. Additionally, as negative values are almost exclusively associated with lagging-strand genes, gene flipping to the atypical orientation appears to be the best explanation for CP1-based GC skew inversions. Both observations support the validity of our interpretations and methods.

**Fig. 2:**
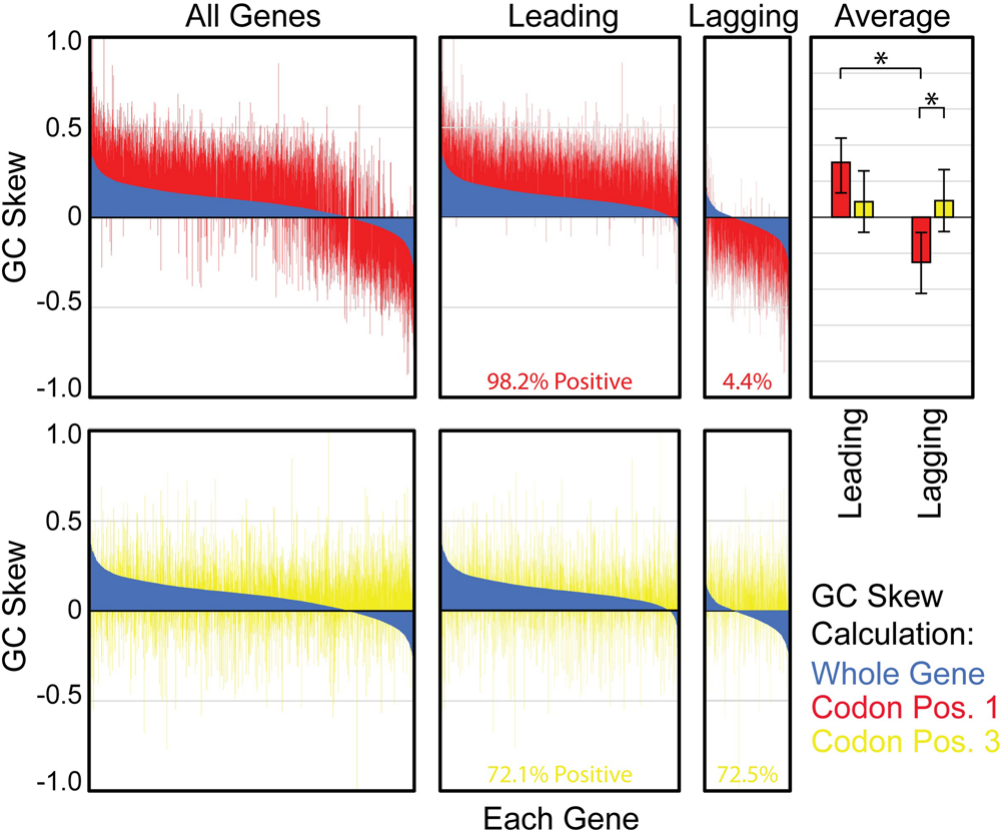
GC Skew values for whole-gene regions. Upper graphs: GC skew values are calculated using whole-gene regions (blue) or only codon position 1 nucleotides (red) for all *B. subtilis* genes, leading strand genes only (Leading), or lagging-strand genes only (Lagging). Genes are sorted based upon the whole-gene GC skew resulting in the appearance of a curve. CP1 and CP3-based values are not independently sorted, allowing for direct comparison between data sets. The average GC skew values (Average) demonstrate that CP1-based GC skew values (red) are significantly different for leading versus lagging-strand genes, whereas CP3-based values (gold) are not. Additionally, CP1-based GC skew values are significantly different from CP3-based values for lagging-strand genes. Error bars represent the standard error of the mean. Significance was determined by the z-test, asterisk symbol (*) indicates *p* = 0.0. The percentage of positive CP1-based GC skew values is shown at the bottom of the Leading and Lagging-strand gene graphs. Lower graphs: Whole-gene (Blue, same data as upper graphs) versus CP3-based GC skew values are shown.

To further test the validity of our method and conclusions, we returned to the hypothesis that the CP3-based GC skew value of a GC skew inversion should rise quickly relative to CP1-based values due to low selective pressure. If this model is correct, then the CP3-based GC skew value of most genes should be higher than the CP1-based value. Indeed, that is exactly what we observed (Fig. 2, gold versus red columns). This case is particularly clear among lagging-strand genes where the average CP3-based value is positive while the average CP1-based value remains negative. These differences are highly significant (Z-test *p* = 0). Notably, these data imply that a CP1-based GC skew analysis should be more accurate than a whole-gene based analysis for detecting genes that change orientation. Therefore, we re-calculated the GC skew values for *B. subtilis* genes using the CP1-based method (Fig. 2 red versus blue columns). Incredibly, the new data indicate that nearly all (94%) of the genes currently on the lagging-strand were encoded on the leading strand throughout the majority of *B. subtilis*’ evolutionary history. This exceeds our original estimate of 64%^4^. As such, our original data appears to be generally accurate, if limited in resolution (discussed below).

Though most of the differences between the GC skew and phylogeny-based data sets are due to GC skew reversions, some discrepancies remain. As one possible explanation, we hypothesized that some genes may have changed orientation in the distant past. In such cases, subsequent mutagenesis could have erased the negative GC skew value, preventing detection of the flip. In keeping with this notion, previous work shows that the closest related ancestor/descendant pair, *Mycoplasma penetrans* and *Mycoplasma gallisepticum*, diverged at least 100M years ago^6^. This is based on a 1.8% difference in the 16S rRNA genes between the ancestor and descendant species, and a 1% rate of change per 50M years^6^. In further support of this temporal limitation hypothesis, we observed that the higher-resolution CP1-based GC skew analysis solved the problem: For the *B. lichenformis*/*B. subtilis* pair, a CP1-based GC skew sign change was apparent in 100% of the flipped ortholog pairs (Supplementary Data 1, right columns). To ensure that this observation does not reflect a high false positive rate, we calculated the CP1 GC skew for all single copy orthologs that did not change orientation (Supplementary Data 5). In this analysis, the phylogeny-based and GC Skew-based analyses show 98.4% agreement. As the remaining discrepancies could reflect an error in either method^7^, our data imply a false negative rate of <1%, and false positive rate of <1.6%. These observations strongly support the validity of the CP1-based GC skew-based analysis. They also suggest that our original (whole-gene) method has a lower temporal limitation that reduces detection of ancient GC skew inversions.

Liu and Zhang also challenge our inference that positive selection acts more frequently on lagging-strand genes. To further test our interpretation, we conducted likelihood ratio tests using the single-nonsynonymous rate models M1a (neutral evolution) and M2a (positive selection) (Fig. 3)^8^. We found that lagging-strand genes with a dN/dS value >1 are likely to be under positive selection (Chi square test *p* < 0.05) ~2.5 times more frequently than leading strand genes (Fisher’s exact test *p* = 0.014) in *M. tuberculosis*. We observe a similar ratio in *B. subtilis*, though the difference between the leading and lagging-strand genes does not reach 95% significance (Fig. 3, middle). We also repeated our previous cross-species analysis, finding that genes with a dN/dS ratio greater than 1 (Chi square *p* < 0.05) that better fit the M2a model are more frequently observed in lagging-strand genes (Fisher’s exact test *p* = 0.005, Fig. 3, right). At the 95% confidence level, we could expect to observe roughly 5 false positive data points among either the 108 leading or 97 lagging-strand genes with a dN/dS >1. As we observe 19 and 26, respectively, our results are well above background. Therefore, these data suggest that our inference of more frequent positive selection on lagging-strand genes was accurate.

**Fig. 3:**
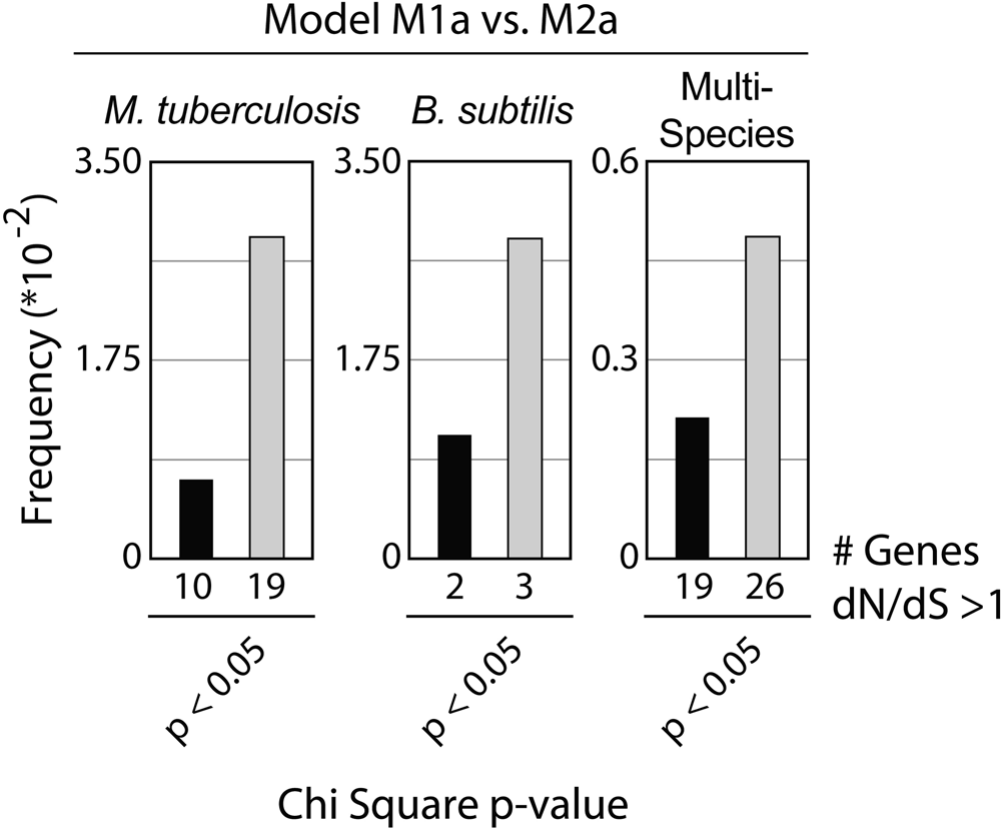
Lagging-strand genes are more frequently under positive selection. Likelihood ratio tests (LRTs) were used to compare site models M1a (neutral model) versus M2a (positive selection model) as a test for positive selection among genes with a dN/dS ratio >1. The frequency of genes better fitting the positive selection model at >95% significance are plotted (# genes under positive selection/total). Positive selection likely acts more frequently on lagging-strand genes in *M. tuberculosis* (Fisher’s exact test *p* = 0.049). An equivalent analysis is shown for *B. subtilis* (middle graph, Fisher’s exact test *p* = 0.12 for leading versus lagging-strand frequencies). Multi-species analysis: across species, genes under positive selection are more frequent in lagging-strand genes, confirming the results of our original analysis (right graph, Fisher’s exact test *p* = 0.005).

Liu and Zhang also claim that the equivalent dS of leading and lagging-strand genes indicate that lagging-strand genes do not mutate at a faster rate, and that this metric contradicts the adaptive hypothesis. This is an interesting point as the dS values in our manuscript are indeed equal for leading and lagging-strand genes. However, the manuscripts Liu and Zhang cite directly contradict their claims: both Schroeder et al.^9^ and Sankar et al.^10^ reported a higher base substitution rate in lagging-strand genes (their Figs. 3A, 1D, respectively). Likewise, a recent study confirmed the higher mutation rate of lagging-strand genes in *E. coli*^11^. Additionally, there are other interpretations of the equal dS values that do not contradict our model. The dS represents a long-term average mutation rate but is uninformative about short-term variations in spontaneous mutation rates. Our work shows that short-term variations are a critical concern. We showed that, when transcribed, lagging-strand alleles have a higher mutation rate than otherwise identical leading strand alleles^1,2^. If leading and lagging-strand genes were equivalent (expression pattern, gene length, etc.), Liu and Zhang’s interpretation would be reasonable. However, there are major differences between these groups^4,12,13^. In Supplementary Fig. 1 (top), we show how a leading strand gene and a (distinct) lagging-strand gene could accumulate a similar number of mutations over the same time period, while having different mutation rates when transcribed. These profiles are based on mutation rates measured using Luria-Delbrück fluctuation assays^1,2^. Here, the lagging-strand allele has twice the mutation rate of the leading strand gene when transcribed, but an equal mutation rate when transcriptionally repressed (Supplementary Fig. 1, top). This model predicts that transcriptional induction is a major factor affecting the dS. We also demonstrate how a flipping event will alter the same gene’s mutation rate under an identical induction profile (Supplementary Fig. 1, bottom). Together, these models reconcile the equal dS observed in nature with the higher spontaneous mutation rate of lagging-strand alleles observed in highly controlled laboratory experiments.

Liu and Zhang further propose that a gene’s GC skew value can become negative due to causes other than gene flipping (GC skew inversion). If correct, this would indeed undermine the validity of our method and conclusions. In support of this notion, Liu and Zhang reference Chen et al.^14^ who showed that transcription, translation, and replication-based nucleotide synthesis cost biases can affect GC and AT skews. Their data suggest that cost bias can increase a gene’s GC skew with respect to the sense strand, resulting in negative GC skew values in lagging-strand genes without physically flipping^14^. This is testable. If the Chen et al.^14^ model is correct, cost bias should cause the more mutable CP3-based GC skew values of lagging-strand genes to be lower than the CP1-based values. If our model is correct, DNA replication should universally drive the CP3-based values higher than the CP1-based values in lagging (and leading) strand genes. Our analysis in Fig. 2 (gold versus red columns) clearly demonstrates that the average CP3-based value (gold) is significantly higher than the CP1-based value (red) in *B. subtilis’* lagging-strand genes (Z-test, *p* = 0). CP3-based values also tend to be positive in absolute terms (Fig. 2, Averages). Both observations strongly support our model. However, Chen et al. observed that low GC organisms (e.g., *B. subtilis*) show increased “indifference” toward amino acid related selection^14^. Therefore, we performed the same test in *M. tuberculosis* (strain H37Rv) which has a high GC content. Again, we observed the same pattern: Among the total 1579 lagging-strand coding genes, 97% (1531 genes) of CP1-based GC skew values are negative or equal to zero, versus 2% for leading strand genes. We also observe that 81% of lagging-strand genes have a positive CP3-based GC skew value. Accordingly, CP3-based GC skew values are higher than the CP1-based value for 95.6% (1510/1579 genes) of lagging-strand genes. Even in *E. coli,* which has a low strand bias^4^, 89% (1759/1978 genes) of lagging-strand genes have a negative CP1-based GC skew value. Among them, the CP3-based GC skew is higher than the CP1-based GC skew in 83% (1463/1759 genes), and 48% (848/1759 genes) of the values are positive. Though this does not contradict findings of Chen et al.^14^ the data strongly suggest that the net effect of all mutational pressures causes the negative GC skew values of lagging-strand genes to increase, equilibrating at a positive value. This strongly suggests that replication-related mutational pressure is the primary factor determining GC skew values, addressing a long-standing debate in the field^15–17^. By extension, gene flipping events appear to be the primary cause of negative GC skew values.

In summary, we have provided a variety of evidence indicating that our original GC skew-based gene inversion analysis was generally accurate^4^. After addressing the semantic conflict over the term “inversion”, and introducing the concept of “GC skew reversions”, it became clear that Liu and Zhang’s inversion (i.e., flipping) data largely agrees with our original GC skew inversion data. Most of the false negative errors Liu and Zhang identified in our analysis simply represent GC skew reversions which we did not attempt to identify. However, some disputed data points were not explainable as reversions. These genes appear to have flipped so long ago (potentially 100M years ago) that subsequent mutagenesis prevented their detection via the whole-gene GC skew method. These can be considered false negative data points in our analysis^4^. However, the positive GC skew of these genes implies that they eventually acclimatized to the new orientation. Under this perspective, they are no longer in an “inverted” state in an absolute sense (our original usage^4^), and are therefore not false negatives. Either way, we largely resolved disparities between Liu and Zhang’s data and our own by improving the GC skew method. This new CP1-based analysis accurately identified an opposing GC skew sign (±) in 100% of the ortholog flips in Liu and Zhang’s *B. licheniformis*/*B. subtilis* comparison, confirming its accuracy over a roughly 100M year divergence time. This higher-resolution method also allowed us to determine that the patterns we originally identified^4^ appear to be far stronger than we initially appreciated. Incredibly, nearly all lagging-strand genes, in bacterial species across the evolutionary tree, may have originally been encoded on the leading strand.

Regarding our inference of increased positive selection on lagging-strand genes, we have provided a new statistical analysis of our original dN/dS data. These results also support our original conclusion and the adaptive hypothesis. This is consistent with the observation that convergent mutations, a second indicator of positive selection, also appear to be more common among lagging-strand genes^1,18^. Importantly, Liu and Zhang’s mutation-selection hypothesis cannot explain the convergent evolution data or the higher frequency genes under positive selection on the lagging-strand. As such, it appears that lagging-strand encoding is adaptive for a subset of current lagging-strand genes. Critically, these models are not mutually exclusive. We propose that a combination of neutral evolution and negative selection against highly transcribed lagging-strand alleles (the mutation-selection hypothesis), as well as positive selection on a subset of lagging-strand alleles, drive the organization of the lagging-strand.

## Methods

### Inference of positive selection

We first compiled the nucleotide sequence alignment files produced by TimeZone v.1 and published in Ref. ^4^. Alignments for genes determined to have a dN/dS value greater than 1 were input into PAML’s CodeML application. Likeliood ratio tests of models M1a (null hypothesis) versus M2a (positive selection) were used to determine if each gene is likely under positive selection at the 95% significance level. CodeML settings were based on established standards^19^. The fraction of all leading or all lagging-strand genes inferred to be under positive selection, and also having a dN/dS ratio >1 were calculated. Only genes fulfilling both criteria were considered to be under positive selection in our analysis. Fisher’s exact test was used to determine if the observed frequencies are significantly different for leading versus lagging-strand genes.

### GC skew calculation method comparison

The GC Skew, defined as (G − C)/(G + C), was calculated for the leading strand sequence of whole-gene regions or using only nucleotides corresponding to the 1st, 2nd, or 3rd codon positions. Python scripts are publicly available at https://github.com/The1stMartian/GCskewAnalyzer. The Pearson Correlation coefficient was calculated for the whole-gene average method versus the indicated codon position-specific method (Supplementary Data 4).

### Independent phylogeny-based analysis vs. GC skew analysis

Data are presented in Supplementary Data 5. The program OrthoFinder 1.0 was used to identify all single copy orthologs between *B. subtilis* strain 168 and *B. licheniformis* strain ATCC 14580 using default settings. Gene orientations were annotated in Microsoft Excel based upon the replication origins and termini listed on the DoriC database^5,20^. Codon position-based GC skew values were calculated using custom Python scripts.

### Reporting summary

Further information on research design is available in the Nature Research Reporting Summary linked to this article.

## Supplementary information

Supplementary Information

Description of Additional Supplementary Files

Supplementary Data 1-5

Reporting Summary

## Data Availability

The code used in this paper is available at https://github.com/The1stMartian/GCskewAnalyzer, and all data are available upon request.
