# Supplementary material for: Reply to: Testing the adaptive hypothesis of lagging-strand encoding in bacterial genomes
Source: Nat Commun. 2022 May 12;13:2627. doi: 10.1038/s41467-022-30014-2 (PMC9098457; doi:10.1038/s41467-022-30014-2)
Supplement: Supplementary file 1 — Supplementary Information [file 41467_2022_30014_MOESM1_ESM.pdf]

Supplementary Information

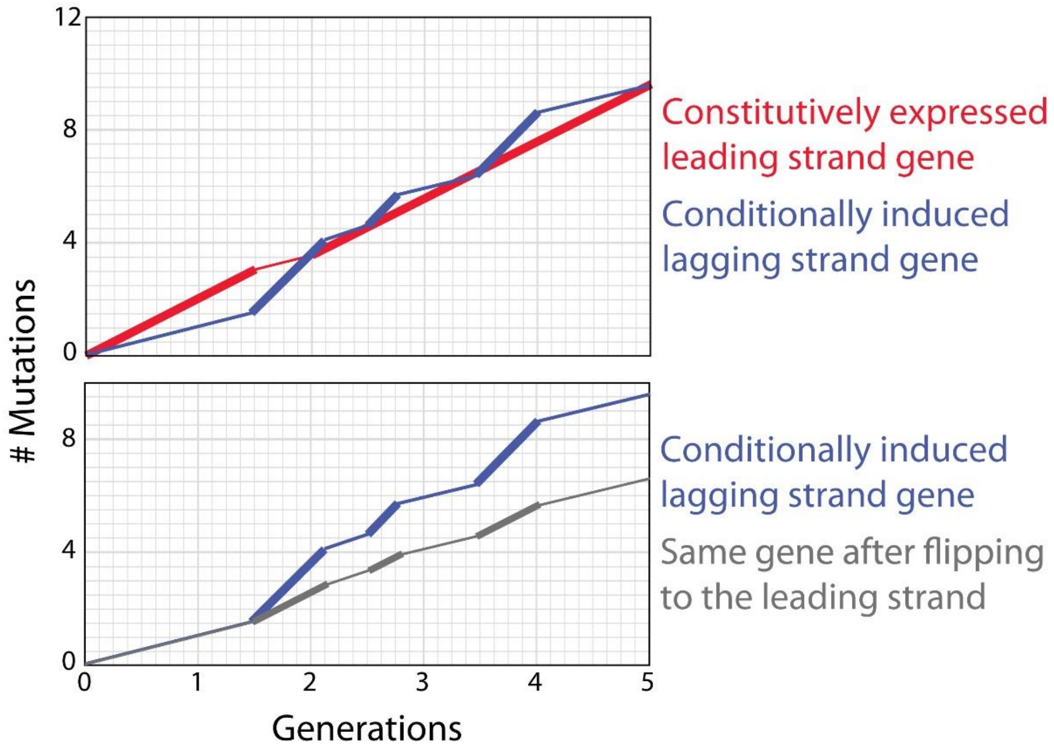

Supplementary Figure 1. Lagging strand genes can have both a higher mutation rate when transcribed, and a dS value equivalent to leading strand genes. Upper graph: Comparison of mutation rates for a hypothetical leading strand gene versus a hypothetical (but distinct) lagging strand gene. The two genes depicted have distinct transcriptional induction patterns consistent with observed trends: the leading strand gene is almost always expressed, whereas the lagging strand gene is briefly induced (for 3 short time periods in this example). On a conceptual level, these differences are consistent with patterns commonly observed for genes in each group[16,17]. The number of spontaneous mutations (a proxy for the dS) for each gene is graphed (leading strand gene in red, lagging strand gene in blue). For each gene, thick lines indicate transcriptional induction, and thin lines represent times of transcriptional repression. Mutation rates (the slope of the lines) change depending upon transcriptional activation/repression as previously described, both generally, and for multiple leading vs. lagging strand reporter genes[8,11,18–20]. Here the leading strand gene (Red) gains mutations at a rate of 2 mutations/generation when transcribed, whereas the lagging strand gene (Blue) gains mutations at 4 mutations/generation when transcribed, as observed for the *hisC952* gene[8]. Both genes have a lower and equivalent mutation rate when transcriptionally repressed (1 mutation/generation). These data show that a similar *average* mutation rate (i.e. dS) can be produced by the two genes, despite the higher mutation rate of the lagging strand gene during transcriptional induction. Lower graph: Mutagenesis patterns for the *same* gene when encoded on in the leading versus the laggings strand. This comparison

isolates the effects of gene orientation, showing that lagging strand encoding yields a higher mutation rate for the same gene consistent with at least 3 reporter genes[8]. Together the upper and lower graphs can explain how lagging strand encoding increases the mutation rate of individual genes, even while ensemble analyses display equivalent average mutation rates (dS values) for leading and lagging strand genes as a group.
