# Supplementary material for: Reply to: Testing the adaptive hypothesis of lagging-strand encoding in bacterial genomes
Source: Nat Commun. 2022 May 12;13:2627. doi: 10.1038/s41467-022-30014-2 (PMC9098457; doi:10.1038/s41467-022-30014-2)
Supplement: Supplementary file 2 — Description of Additional Supplementary Files [file 41467_2022_30014_MOESM2_ESM.pdf]

Description of Additional Supplementary Files

File Name: Supplementary Data 1
Description: Merrikh re-analysis of Liu and Zhang's B. subtilis data

File Name: Supplementary Data 2
Description: Merrikh re-analysis of Liu and Zhang's M. gallisepticum data

File Name: Supplementary Data 3
Description: Merrikh re-analysis of Liu and Zhang's S. aureus data

File Name: Supplementary Data 4
Description: The GC skew calculation yields different results depending upon the method used. The GC skew value for every gene was calculated using either the whole gene sequence or only nucleotides in the 1st, 2nd, or 3rd codon positions. The Pearson correlation coefficients between the codon position-based data sets (indicated on the left) and the whole-gene data based set were calculated and binned by strand for the indicated species.

File Name: Supplementary Data 5
Description: **Confusion matrix of GC skew versus Phylogeny-Based Analyses.** Based on the results of a phylogeny-based analysis of B. subtilis 168 and its ancestor B. lichenformis, B. subtilis genes were binned into two columns: Changed Orientation or No Change. The codon position 1-based calculation was then used to determine if the same genes also appear to have changed orientation according to the GC skew method. Gene pairs (ancestral versus descendant) that have the same sign (either positive in both or negative in both) are interpreted as having remained in the same orientation (no change). Gene pairs with opposing signs are interpreted as having changed orientation. Type I errors are highlighted in blue, Type II errors are highlighted in red.
