# Supplementary material for: Reply to: Testing the adaptive hypothesis of lagging-strand encoding in bacterial genomes
Source: Nat Commun. 2022 May 12;13:2627. doi: 10.1038/s41467-022-30014-2 (PMC9098457; doi:10.1038/s41467-022-30014-2)
Supplement: Supplementary file 4 — Reporting Summary [file 41467_2022_30014_MOESM4_ESM.pdf]

nature portfolio

Corresponding author(s): Houra Merrikh and Christopher Merrikh

Last updated by author(s): 2/16/2022

Reporting Summary

Nature Portfolio wishes to improve the reproducibility of the work that we publish. This form provides structure for consistency and transparency in reporting. For further information on Nature Portfolio policies, see our Editorial Policies and the Editorial Policy Checklist.

Statistics

For all statistical analyses, confirm that the following items are present in the figure legend, table legend, main text, or Methods section.

n/a	Confirmed	
☐	☒	The exact sample size (*n*) for each experimental group/condition, given as a discrete number and unit of measurement
☐	☒	A statement on whether measurements were taken from distinct samples or whether the same sample was measured repeatedly
☐	☒	The statistical test(s) used AND whether they are one- or two-sided<br>*Only common tests should be described solely by name; describe more complex techniques in the Methods section.*
☒	☐	A description of all covariates tested
☒	☐	A description of any assumptions or corrections, such as tests of normality and adjustment for multiple comparisons
☐	☒	A full description of the statistical parameters including central tendency (e.g. means) or other basic estimates (e.g. regression coefficient) AND variation (e.g. standard deviation) or associated estimates of uncertainty (e.g. confidence intervals)
☐	☒	For null hypothesis testing, the test statistic (e.g. F, t, r) with confidence intervals, effect sizes, degrees of freedom and P value noted<br>*Give P values as exact values whenever suitable.*
☒	☐	For Bayesian analysis, information on the choice of priors and Markov chain Monte Carlo settings
☒	☐	For hierarchical and complex designs, identification of the appropriate level for tests and full reporting of outcomes
☒	☐	Estimates of effect sizes (e.g. Cohen's *d*, Pearson's *r*), indicating how they were calculated

Our web collection on statistics for biologists contains articles on many of the points above.

Software and code

Policy information about availability of computer code

Data collection	We used the CodeML application of PAML v.4.8 to calculate dN/dS values
Data analysis	We used Microsoft 356's Excel application to calculate frequencies, quantpsy.org's Fisher's Exact Test and Chi squared tool to calculate significance. GC Skew analysis was performed using the custom GCskewAnalyzer python script version 7 (GCS_v7.py). The program OrthoFinder was used to identify conserved orthologues between species.

For manuscripts utilizing custom algorithms or software that are central to the research but not yet described in published literature, software must be made available to editors and reviewers. We strongly encourage code deposition in a community repository (e.g. GitHub). See the Nature Portfolio guidelines for submitting code & software for further information.

Data

Policy information about availability of data

All manuscripts must include a data availability statement. This statement should provide the following information, where applicable:

- Accession codes, unique identifiers, or web links for publicly available datasets
- A description of any restrictions on data availability
- For clinical datasets or third party data, please ensure that the statement adheres to our policy

All data and code are available upon request. Custom Python scripts for GC Skew analysis are available at https://github.com/The1stMartian/GCskewAnalyzer. OrthoFinder can be downloaded from https://github.com/davidemms/OrthoFinder.

Field-specific reporting

Please select the one below that is the best fit for your research. If you are not sure, read the appropriate sections before making your selection.

- [x] Life sciences
- [ ] Behavioural & social sciences
- [ ] Ecological, evolutionary & environmental sciences

For a reference copy of the document with all sections, see nature.com/documents/nr-reporting-summary-flat.pdf

Life sciences study design

All studies must disclose on these points even when the disclosure is negative.

Sample size	Sample size determination: In our comparative analyses we examined highly conserved orthologues shared by an ancestral and descendant species. The number of genes identified by OrthoFinder under standard settings determined the sample size in this case. For our genome-wide analyses, the number of genes in each genome determined the sample size (typically around 4000 genes per species).
Data exclusions	None
Replication	To determine if the patterns we observed are reproducable, we examined multiple bacterial species across the evolutionary tree. These included B. subtilis, a gram-negative Fermicute, M. tuberculosis which is part of a distinct phylum, and E. coli which is distantly related to the other two species. All three species are separated from one another by millions of years of evolution.
Randomization	None
Blinding	None

Reporting for specific materials, systems and methods

We require information from authors about some types of materials, experimental systems and methods used in many studies. Here, indicate whether each material, system or method listed is relevant to your study. If you are not sure if a list item applies to your research, read the appropriate section before selecting a response.

Materials & experimental systems

n/a	Involved in the study
[x]	[ ] Antibodies
[x]	[ ] Eukaryotic cell lines
[x]	[ ] Palaeontology and archaeology
[x]	[ ] Animals and other organisms
[x]	[ ] Human research participants
[x]	[ ] Clinical data
[x]	[ ] Dual use research of concern

Methods

n/a	Involved in the study
[x]	[ ] ChIP-seq
[x]	[ ] Flow cytometry
[x]	[ ] MRI-based neuroimaging
